# A Novel Wearable Electronic Nose for Healthcare Based on Flexible Printed Chemical Sensor Array

**DOI:** 10.3390/s141019700

**Published:** 2014-10-22

**Authors:** Panida Lorwongtragool, Enrico Sowade, Natthapol Watthanawisuth, Reinhard R. Baumann, Teerakiat Kerdcharoen

**Affiliations:** 1 Faculty of Science and Technology, Rajamangala University of Technology Suvarnabhumi, Nonthaburi 11000, Thailand; E-Mail: dang_phy@hotmail.com; 2 Department of Digital Printing and Imaging Technology, TU Chemnitz, Chemnitz 09126, Germany; E-Mails: enrico.sowade@mb.tu-chemnitz.de (E.S.); reinhard.baumann@mb.tu-chemnitz.de (R.R.B.); 3 Nanoelectronic and MEMS Lab National Electronic and Computer Technology Center, Pathumthani 12120, Thailand; E-Mail: thesaboo@gmail.com; 4 Department Printed Functionalities, Fraunhofer ENAS, Chemnitz 09126, Germany; 5 Department of Physics and NANOTEC's Center of Excellence, Faculty of Science, Mahidol University, Bangkok 10400, Thailand

**Keywords:** armpit, body odor, composite materials, electronic nose, healthcare monitoring, inkjet-printing technology, wearable device, ZigBee, wireless device

## Abstract

A novel wearable electronic nose for armpit odor analysis is proposed by using a low-cost chemical sensor array integrated in a ZigBee wireless communication system. We report the development of a carbon nanotubes (CNTs)/polymer sensor array based on inkjet printing technology. With this technique both composite-like layer and actual composite film of CNTs/polymer were prepared as sensing layers for the chemical sensor array. The sensor array can response to a variety of complex odors and is installed in a prototype of wearable e-nose for monitoring the axillary odor released from human body. The wearable e-nose allows the classification of different armpit odors and the amount of the volatiles released as a function of level of skin hygiene upon different activities.

## 1. Introduction

Due to promising applications such as healthcare, sport and security, investment in the research and development of wearable monitoring systems have been growing rapidly. Specifically, wearable electronic noses (e-noses) have recently been of interest in the healthcare industry as it may help to promote personal healthcare applications together with other well-settled wearable devices such as smart watches and smart shoes. Wearable e-nose can be used as a physiological measurement system for tracking of real-time health status or body hygiene that also allows for long-term analysis [1–3].

According to literature, numerous researches have recently been conducted to investigate health status or unusual conditions of individuals by means of body odor monitoring. This is because of the nature of the odors released from the human body that contain information about biophysical characteristics of individuals such as behavior, emotional state, age, gender, and health status [4,5]. Therefore, the ability to monitor, analyze and correlate these odors to the health information will lead to new and novel biomedical devices with a market of unprecedented size. For instances, García-Cortés *et al.* [6] reported about using an e-nose to detect the stress stages through sweat analysis. They studied alert situations that correlate with stress and have mentioned about the secretion level of cortisol and adrenaline hormones in each stage. Other interesting research works include using e-noses to diagnose lung cancer based on breath analysis [7], identify heart failure based on skin odor detection [8], and determine the patients with kidney disorders through urine odor measurement [9]. Besides the interest to use body odor as an indicator for personal health status, the potential to use this property to identify people (biometrics) was also explored. Inspired by the ability of dogs to memorize owners and acquaintances by sniffing their body odor, Wongchoosuk *et al.*, proposed a scheme to discriminate two people living a normal life based on measurement of daily armpit odors [10]. From this report, it was shown that the armpit odor of an individual can significantly vary due to daily activities but the difference between two persons can still be identified.

As a matter of fact, the above-mentioned research works handle the body odor samples for off-line detection, thereby lacking of a real-time functionality. Moreover, with the working principle of the common e-noses, they are also considered as promising candidates for the rapid screening technique to evaluate the odors in different applications [11,12].

In the present work, we propose a new scheme that can measure fresh and native odors right away from the body, e.g., under the armpit, thus allowing the device to track real-time and dynamic change of the body odor of humans. Under this scheme, a gas sensor array sensitive to the body odor has been fabricated as a wearable device using inkjet printing technology. CNTs/polymer composites were chosen as sensing materials according to their promising working conditions suitable for wearable system, *i.e.*, operating at low-temperature with low-power consumption. The electrical response of these chemical sensors can be explained by the behavior of polymer swelling upon penetration of volatiles or gases into the sub-surface of the CNTs/polymer film. This increases the distance between the conducting pathways of CNTs and thus causing change in the electrical resistance of the sensor [13]. Physical properties of the blended CNTs in the polymer matrix allow the fabrication on flexible substrates and thus suitability for wearable applications. With the solution-based preparation of CNTs/polymer composite layers, the chemical sensors can be possibly carried out by simple methods such as drop-casting, spraying, spin-coating or printing which are cheaper and simpler in comparison to methods based on evaporation. Among these solution-based methods, the materials deposited by printing prevail over other techniques due to film uniformity, precise deposition on a desired position and scalability [14].

In this paper, we investigate the feasibility of inkjet-printing for the manufacturing of low-cost sensors. This technology allows the dispensing of functional materials onto a small, pre-determined area, processing under ambient conditions and also reducing material waste since it is an additive direct-writing technology [15]. Moreover, the low operating temperature of the inkjet-printing method is suitable for the deposition of organic materials onto common flexible substrates such as polyethylene naphthalate (PEN), polyethylene terephthalate (PET), polyimide (PI), paper or fabric [16–19] in which such materials are easily decomposed at a high temperature. Beside the fully printed sensor array, we have also reported about the architecture of a novel wearable e-nose system using a ZigBee module for wireless network system to monitor the armpit odor.

## 2. Experimental Section

### 2.1. Fabrication of Chemical Sensor Array

**Figure 1.**
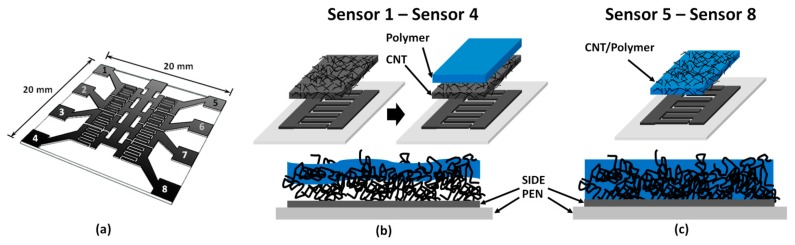
Architectures of (**a**) the silver interdigitated electrode (SIDE); (**b**) the double printed layers (DPLs) sensor and (**c**) the blended single layer (BSL) sensor.

The chemical sensor array was fabricated by fully inkjet-printing technique. The array comprises eight different elements produced by varying printing patterns and sensing materials which are able to generate the cross responses [20–23]. Polyethylene naphthalate (PEN film, Dupont Teonex Q65FA) film was chosen as substrate material due to its flexible property, smooth surface and heat stability. We have designed the chemical sensor array into two sets. The fabrication scheme of an individual pattern is depicted in Figure 1.

The first set (Sensor 1 to Sensor 4) is called as “double printed layers” (DPLs) as illustrated in Figure 1b. Thereby, two individual films of CNTs and polymer were printed to produce a composite-like layer at the interface. This technique can neglect the challenges concerning the development of well-dispersed formulation of CNT-polymer ink solution [19]. For the second set (Sensor 5 to Sensor 8), the sensing film was produced by direct printing of the blended CNT-polymer ink to obtain the so-called “blended single layer” (BSL) as shown in Figure 1c. The idea and advantage of printing using different patterns was reported in our previous work [24,25]. To fabricate each of the DPLs and BSL sensors, we used multi-walled carbon nanotubes (MWCNTs) and four polymers; namely (i) polyvinyl chloride (PVC); (ii) cumene terminated polystyrene-co- maleic anhydride (Cumene-PSMA); (iii) poly (styrene-co-maleic acid) partial isobutyl/methyl mixed ester (PSE) and (iv) polyvinylpyrrolidon (PVP). Multi-walled carbon nanotubes (MWCNTs) were purchased from Southwest Nanotechnologies. All polymers and other materials were obtained from Sigma-Aldrich. MWCNTs and polymer inks need to be separately prepared for construction of the DPLs sensors, whereas for the BSL sensors it requires only one MWCNTs/polymer composited ink. The MWCNT ink was prepared by adding 0.2 wt % MWCNTs into an aqueous solution of 0.5 wt % sodium dodecyl sulfate (SDS). High frequency pulses of the ultrasonic homogenizer (Bandelin, model HD 3200) were applied for 15 min to disperse and unbundle the MWCNTs. After the black solution was obtained, the remaining large particles were separated by centrifugation for 20 min at 20,000 rpm and then only the upper part of the solution was collected. For the polymer ink, 0.5 wt % of each polymer was simply prepared in the proper solvent by applying a magnetic stirrer for 10 min. The ink for construction of the BSL sensor was obtained by blending of 0.1 wt % MWCNTs, 0.5 wt % polymer and 0.5 wt % surfactant material in the proper solvent. Mixing procedure was performed in the same way as preparation of the MWCNTs ink. The surfactant materials used to stabilize the MWCNTs dispersion in the polymer solution are Triton X-100 for MWCNTs/PVC and MWCNTs/Cumene-PSMA inks, and SDS for MWCNTs/PSE and MWNTs/PVP. However, the prepared inks should be filtered at the final step using 5 μm pore-sized disposable syringe filter prior to inkjet printing.

Each layer was printed for five times to increase the uniformity and the active material amount of the sensing layers and thereby improving the efficiency of the sensing responses. The Dimatix Materials Printer 2831 (DMP) with printheads having a nozzle diameter of 21.5 μm and the Autodrop micro dispensing system (Microdrop Technologies) with a nozzle diameter of about 70 μm were used for printing. Commercially available silver nanoparticle ink (Suntronic SunJet Silver EMD5603) was inkjet-printed as an array of silver interdigitated electrodes (SIDEs) on top of the PEN substrate, followed by printing of the sensing layers. The architecture of an interdigitated electrode array is shown in Figure 1a. In detail, the fingers of each SIDE were defined for the width and separation distance of 200 μm. The size of the array is about 20 mm × 20 mm. The area of sensing material inkjet-printed on top of each SIDE was a square of 3 mm × 3 mm. All sensing layers including polymer, MWCNTs and MWCNTs polymer films were carried out by the Autodrop micro dispensing system. Finally, the printed sensor array was heated to 80 °C for 15 min to remove remaining solvents. The materials and printing parameters for all fabricated sensors are summarized in Table 1.

### 2.2. Testing of the Chemical Sensor Array

**Figure 2.**
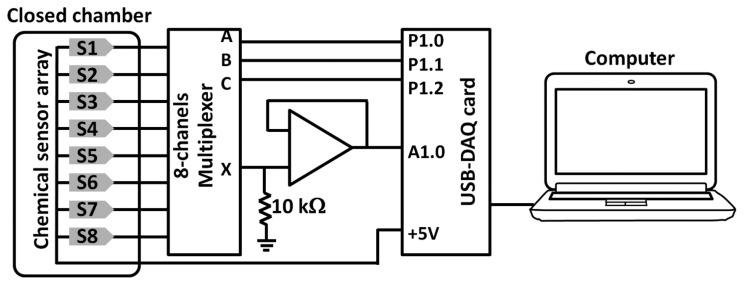
Measurement diagram using a voltage divider method with buffer amplifier for the sensor response under a static system.

Response of the fabricated chemical sensor array to selected volatile organic compounds (VOCs) was investigated under a static system at room temperature. The printed chemical sensor array was installed in a closed chamber and was measured for the resistance change of individual sensor when exposed to a single VOC using voltage divider method. The measurement circuit is shown in Figure 2.

In fact, the VOCs derived from human skin normally contain information about human health (inner factor) and skin hygiene (outer factor). Armpit area presents not only a high density of skin glands (sebaceous, apocrine and eccrine) but also high density of bacteria due to airless and moistness area. Major VOCs from the human skin were identified to contain several compounds such as ammonia, amines, hydrocarbons, alcohols, acids, ketones, and aldehydes [26,27]. Therefore different single VOCs of ammonium hydroxide, acetic acid, acetone and ethanol were chosen to test the sensor array by injection of 500 ppm VOCs in the static chamber.

The output voltages of eight elements were acquired through an 8-channel analog multiplexer connected to a USB DAQ device and then the Ohm's law was employed to calculate individual sensor resistances. To obtain the sensing response to each volatile, the reference baseline was defined during the first 2 min without exposure to any VOCs (ambient condition). Then, the analyte was injected into the static chamber and the output signals were recorded for 7 min to reach a steady-state condition. The fractional method was applied on the determined sensor resistances to calculate the sensor responses as follows [28]:

(1) $$\%\text{Sensor response}=\frac{R_{s}-R_{o}}{R_{o}}\times 100$$

where *R_o_* is the reference resistance of sensor under an ambient condition, and *R_s_* is the sensor resistance at a steady-state condition after injection of analyte gas into the static chamber.

### 2.3. Wearable e-Nose Based on ZigBee Wireless Network

**Figure 3.**
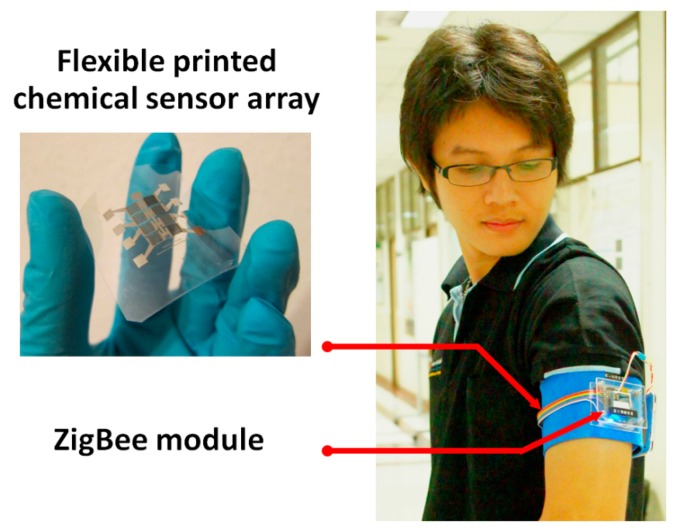
Prototype of a wearable e-nose based on using the flexible inkjet-printed chemical sensor array integrated in ZigBee wireless network.

We have designed a compact armband appropriate for monitoring the axillary odor by integration of the flexible printed chemical sensor array. Figure 3 depicts a prototype of the designed wearable e-nose. The printed sensor array covered by a mask was placed at the armpit region. The mask will protect the sensor surface from scratching but allow passing of the air and odors. With this wearable device, the body odor could be directly collected from the armpit region. The odor data was recorded in terms of the sensor resistances as a function of the presented VOCs and their concentrations. ZigBee technology, widely known as a low-cost wireless network communication, was employed in the system to transfer the data from the device to the computer. This technology also allows monitoring in real time and an increasing number of nodes for network communication which will be planned in the future. Within this module, the ZigBee system supports the multi-hop mesh network topology which enhances the reliability and network stability.

**Figure 4.**
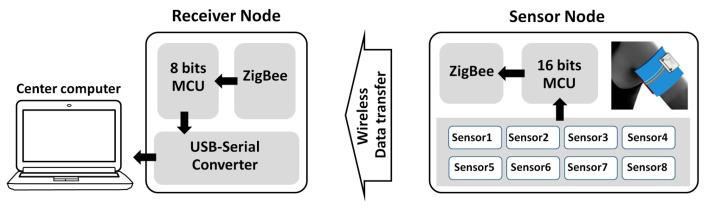
Working principle of the wearable e-nose based on ZigBee wireless network communication.

The Xbee-PRO RF module based on ZigBee/IEEE 802.15.4 was employed for the wireless network. This technology offers advantages in cost, size, power, flexibility and distributed intelligence as compared to the wired technologies. The ZigBee module consists of two main nodes, *i.e.*, sensor and receiver nodes as depicted in Figure 4. In the sensor node, the fabricated chemical sensor array was installed to sense the volatile odors while a 16-bit microcontroller unit (MCU) from Microchip (PIC24HJ12GP201) was used as a control unit for the sensor node. The 16-bit MCU reads voltage indicator from the sensors via ADC port, then transferring the obtained data to the receiver node via ZigBee wireless network. Another node is the receiver node system. The system consists of ZigBee, 8-bit MCU, and a USB-serial converter chip. The receiver node receives data and transfers them to a computer via a USB port.

### 2.4. Monitoring of Axillary Odor

Based on a preliminary study, the experiment was designed to monitor armpit odors of three volunteers (33–50 years-old healthy subjects) using the constructed wearable e-nose. The odors were monitored by following activities of the volunteer as simulated in three stages: (i) before exercise (normal stage and used as reference); (ii) after exercise and (iii) after exercise and relaxing for a long time. To perform all experiments, the reference baseline of the sensors was collected by exposing them to the clean air and then followed by each activity. After the volunteer had worn the device, we recorded the odor data for 5 min. The sensor resistances were averaged in every minute and then the sensing responses were calculated to obtain 5 data points per experiment. The experiments were carried out three times for each stage. The reference baseline was recovered before performing the next experiment. Therefore, for one stage of the experiment, we can obtain 15 data points (5 data points × 3 repeating experiments) representing the sensor responses to armpit odors corresponding to the simulated activity. Principal component analysis (PCA) based on the unsupervised learning method was employed to analyze the responsive patterns.

## 3. Results and Discussion

**Figure 5.**
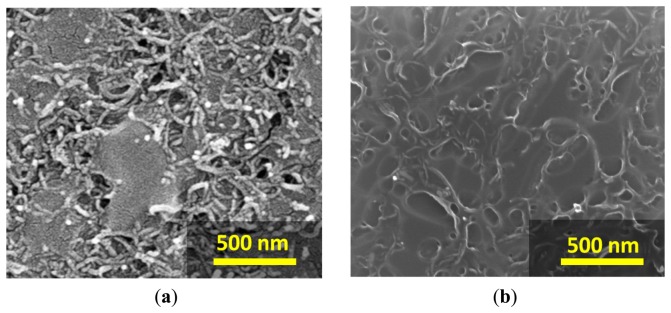
SEM images of surface morphology: (**a**) the DPLs sensor and (**b**) the BSL sensor.

The flexible chemical sensor array was successfully manufactured with inkjet-printing technology as shown in Figure 3. Each element of the array consists of silver interdigitated electrode coated with the sensing film. The sensing unit is based on the flexible substrate, thereby posing an advantage for the wearable device. Moreover, this feature could be considered as an important step to enable roll-to-roll processing in order to scale up the production in the future. We have been focusing on the sensing materials made of polymers and CNTs because they present good physical properties and are suitable for flexible substrates. Especially, they can overcome the metal oxide gas sensor due to the lower operating temperatures [10]. In this work, the composited MWCNTs/polymer films were demonstrated to be produced through inkjet-printing of separated layers between MWCNTs and polymer films (DPLs) and dispensing of the blended solution (BSL). The surface morphologies of both sensor types obtained from scanning electron microscopy (SEM) are displayed in Figure 5.

The MWCNTs films have a good material dispersion and thus forming a conductive network. It was demonstrated that the printed polymer as a second layer on top of MWCNTs surface can penetrate into the MWCNTs network and yield a composite-like layer as shown in Figure 1b. In Figure 5b, the SEM image shows the top-view morphology of the BSL sensor of the inkjet-printed blended MWCNTs/polymer solution.

In the field of the e-nose technology, the variety of sensors is also required in order to employ the advantage of pattern recognition [20]. Therefore, most research about e-noses based on CNTs/polymer sensing materials varies the sensor by using different polymers [29–31]. Although it is convenient to produce different sensors by changing of the sensing materials, printing CNTs/polymer often suffers from agglomeration of CNTs in the polymer matrix leading to nozzle clogging. Therefore, the DPLs sensor printing presents the advantage over the BSL method. With this printing technique we can produce all CNTs/polymer composite layers without any chemical treatment. However, in this work we have integrated both DPLs and BSL sensors into one chemical sensor array. The sensor responses were improved and intensified by printing of multi-layers to increase the amount of sensing material as suggested in the previous works [24].

**Figure 6.**
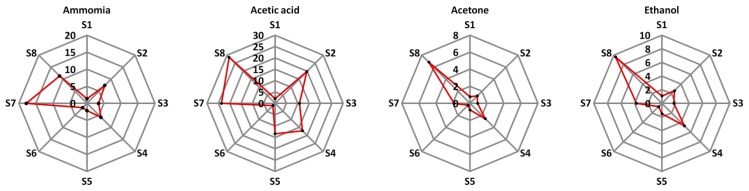
Radar plot of percent sensor response of eight sensor elements (S1–S8) in a static system when exposed to volatiles of ammonia, acetic acid, acetone and ethanol with a concentration of 500 ppm.

Based on the setup in Figure 2, electrical responses of the sensors towards ammonia, acetic acid, acetone and ethanol, which are the class of VOCs derived from axillary skin [26,27], were obtained and shown in Figure 6. Most sensors provide a good response to both acid and base compounds while they show a small change when exposed to ketone and alcohol. According to different printed sensing layers (DPLs and BSL), we have found that the electrical responses of the fabricated chemical sensor array yields distinguishable patterns when exposed to the volatiles of ammonia and acetic acid while the electrical responses under the atmosphere of acetone and ethanol show quite the same patterns.

This may imply that our chemical sensor array is not good enough to classify the difference between acetone and ethanol. However, the major chemical components of body odor were found to contain C6–C10 straight chain, branche, and unsaturated acids [4,32]. In addition, ammonia and other nitrogen compounds were often used to indicate about a health status regarding to the body metabolism [4]. In previous works, we have demonstrated that the sensors based on the polymers of PVC, Cumene-PSMA, PSE and PVP can provide good responses to ammonia and amine volatile compounds [33,34]. Therefore, this fabricated sensor array in the present work is considered as one of the promising candidates for sensing health status as well.

Actually, the number of chemical sensors in an e-nose system is also one of the important factors concerning the capability to classify odors. For example, a commercial e-nose, Cyrano Sciences' Cyranose 320 comprises 32 polymer carbon black composite sensors can be used to predict six classes of bacteria related to eye infections with up to 98% accuracy [31]. In this article, we report the e-nose system comprising only 8 CNTs/polymer sensors to monitor armpit odors of individuals upon different activities. Although our proposed chemical sensors contains 4 times less sensor materials than the Cyranose, the results also demonstrate a good performance of the device.

**Figure 7.**
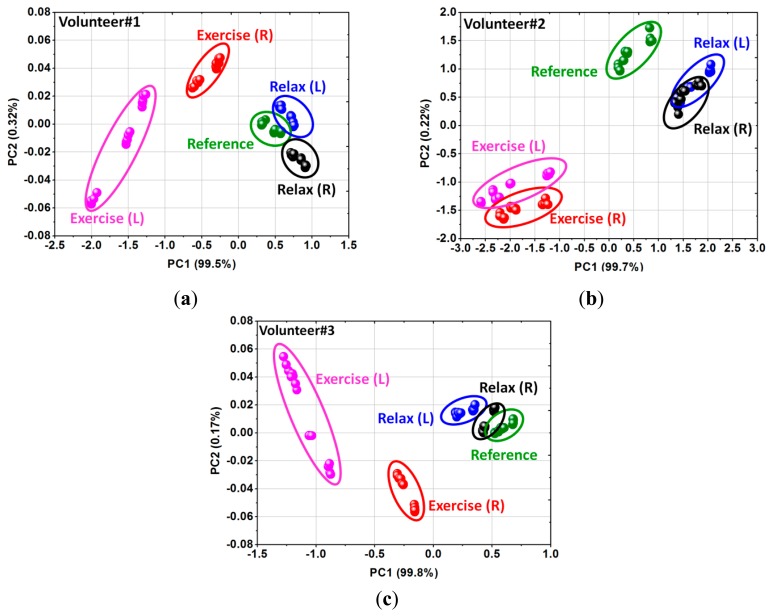
PCA scores plot of underarm odor under different activities: (**a**) volunteer#1; (**b**) volunteer#2 and (**c**) volunteer#3.

Figure 7 shows a classification of the armpit odors under the simulated activities using PCA technique of three volunteers to demonstrate the real-world application. The results can be observed by the variations of principal component scores of all three stages. Odor progression after exercise was monitored and it was found that the data groups were moved out from a normal stage (reference). In addition, we can observe recovering of the underarm odor after relaxing of the volunteer. This result provides a straightforward understanding about the relationship between the observed signals and changing of the human odors related to different activities. According to the corresponding results from three volunteers, it may guarantee that the proposed system is repeatable and able to make real time monitoring of the skin hygiene. In other words, the wearable e-nose could be used to evaluate unusual odors of body resulted of health status, *i.e.* the level of skin hygiene and unusual odors from ailments or stress.

## 4. Conclusions

The wearable e-nose based on a ZigBee wireless network designed as a compact armband shows the feasibility to differentiate the VOCs released from the armpit regions. Based on the principal component analysis, the results can indicate the level of skin hygiene compared to the normal stage. In this work, we demonstrate the successful manufacturing of the flexible chemical sensors array by inkjet-printing technique—a low-cost, flexible and scalable technology. The manufactured eight sensors can respond and generate fingerprints of various VOCs due to different printed patterns and various sensing materials. The sensors with DPLs provide a good idea to implement other composite-sensing layers without direct forming of the CNT/polymer composite. Based on the ZigBee technology, it allows monitoring in the real-time and increasing a number of nodes for network communication which is planned in the future.

## Figures and Tables

**Table 1.** Dispensing parameters of the Autodrop micro dispensing system to deposit the sensing layers.

**Sensor No.**	**Printed Materials**	**Drop Spacing (μm)**	**Printing Speed (mm/s)**	**Substrate Temperature (°C)**
1	(1) MWCNTs	100	2	80
	(2) PVC	100	5	25

2	(1) MWCNTs	100	2	80
	(2) Cumene-PSMA	100	8	25

3	(1) MWCNTs	100	2	80
	(2) PSE	100	10	25

4	(1) MWCNTs	100	2	80
	(2) PVP	100	5	25

5	MWCNTs/PVC	110	5	30

6	MWCNTs/Cumene-PSMA	110	2	85

7	MWCNTs/ PSE	120	15	50

8	MWCNTs/PVP	120	5	80
